# Association between transmission modes and chronic Chagas disease clinical forms

**DOI:** 10.1590/0037-8682-0432-2024

**Published:** 2025-06-16

**Authors:** Carlos Walmyr de Mattos Oliveira, Luiz Henrique Conde Sangenis, Sergio Salles Xavier, Roberto Magalhães Saraiva, Mauro Felippe Felix Mediano, Andrea Silvestre de Sousa, Alejandro Marcel Hasslocher-Moreno

**Affiliations:** 1 Instituto Nacional de Infectologia Evandro Chagas, Fundação Oswaldo Cruz, Rio de Janeiro, RJ, Brasil.; 2 Setor de Radiologia, Hospital do Câncer II, Instituto Nacional de Câncer, Rio de Janeiro, RJ, Brasil.; 3 Centro Universitário de Volta Redonda - UniFOA, Volta Redonda, RJ, Brasil.; 4 Universidade Federal do Rio de Janeiro, Faculdade de Medicina, Rio de Janeiro, RJ, Brasil.

**Keywords:** Chagas disease, Epidemiology, *Trypanosoma cruzi* transmission, Clinical forms

## Abstract

**Background:**

Chagas disease (CD) is associated with significant morbidity and mortality due to cardiac and/or gastrointestinal complications. CD transmission has diverse modes, and their potential relationship with the clinical forms of CD remains unexplored. This study evaluated the association between the transmission modes and chronic clinical forms of CD.

**Methods:**

This retrospective study included patients with chronic CD referred to the outpatient clinic of INI/Fiocruz between November 1986 and August 2024. Clinical and epidemiological data were retrieved from medical records. Sociodemographic profiles, epidemiological history of CD, and clinical, cardiac, and digestive evaluations were assessed. Unadjusted and adjusted logistic regression models were fitted to assess the association between transmission modes and CD clinical forms.

**Results:**

The analysis included 2,162 patients (53.0% women; mean age 48.3 years). The CD transmission mode was evaluated as vectorial in 1962 (90.8%) patients, followed by blood transfusion in 123 (5.7%), congenital in 59 (2.7%), and oral in 18 (0.8%). Patients with congenital or oral transmission were younger, less likely to be women or self-reported as black, had lower rates of illiteracy, and a higher percentage were from non-endemic areas. No significant associations were observed between transmission modes and the cardiac or digestive forms of CD in unadjusted and adjusted analyses.

**Conclusions:**

Vectorial transmission was the most common transmission mode in patients with chronic CD. No significant association was found between the transmission mode and CD clinical forms, indicating that other mechanisms associated with progression to chronic determinate forms should be investigated.

## INTRODUCTION

Chagas disease (CD) is a neglected infectious condition caused by the protozoan *Trypanosoma cruzi* (*T. cruzi*). In 2010, the Pan American Health Organization estimated that approximately 6 to 7 million people worldwide had CD^1^. A study in 2024 updated the prevalence of CD in Brazil to 3.7 million people, with a predominance of cases among women^2^. Currently, CD is present not only in Latin American countries but also in non-endemic regions owing to globalization and migration processes^3^. CD can cause significant morbidity and mortality due to cardiac and gastrointestinal complications, which define the determinate clinical forms of the disease: the cardiac and digestive forms^4^.

The modes of transmission of CD are diverse. Historically, vector-borne transmission has been the primary route of infection, which occurs when individuals are bitten by triatomine insects infected with *T. cruzi*. During the blood meal, the triatomine defecates, and *T. cruzi* present in its feces enters the host's body through the bite wound or the ocular mucosa. Other modes of transmission include blood transfusions or organ transplantation, congenital or vertical transmission, occupational or laboratory accidents, and oral transmission^4^. Recently, the number of cases attributed to oral transmission has increased, highlighting its increasing importance among transmission modes. In the Amazon and other rural areas of Latin America, the consumption of açaí, sugarcane, and acerola juice, along with wild game meat, were identified as potential vehicles for the oral transmission of *T. cruzi*, emphasizing the need for epidemiological surveillance and sanitary control measures in these regions^5^. Hypothetically viable alternative modes, under conditions of high parasitemia in the infected individual, include sexual transmission, breastfeeding, involvement of other types of arthropods, contact with secretions from the anal glands of marsupials, and other practices such as blood pacts in love rituals and the sharing of needles and syringes for injectable drug use^6^. Moreover, the geographical expansion of CD raises concerns regarding outbreaks in areas with limited awareness and inadequate healthcare resources for disease management. As populations migrate, the potential for transmission through blood transfusion, organ transplantation, and congenital routes increases, posing a public health challenge in nonendemic countries^7^ ^,^ ^8^

The relationship between transmission modes and clinical manifestations of CD is complex and multifactorial, involving dynamic interactions between the parasite, host, and environment^9^. Differential transmission routes may lead to different clinical outcomes based on factors such as the parasite load, strain variability, and host immune response. For instance, acute CD acquired through vector bites may result in milder symptoms than with that acquired through oral transmission, probably due to higher parasite loads^10^. However, the potential relationship between the transmission modes and chronic clinical forms of CD remains unexplored. Understanding these dynamics is important to improve prevention strategies and tailor treatment approaches for patients with CD^11^ ^,^ ^12^. In this context, this study aimed to evaluate the association between the transmission modes of CD and its cardiac and digestive clinical forms during its chronic phase.

## METHODS

This retrospective study included patients diagnosed with chronic CD who were referred to the outpatient center of the Evandro Chagas National Institute of Infectious Diseases (INI) of the Oswaldo Cruz Foundation (Fiocruz) between November 1986 and August 2024. Clinical and epidemiological data were retrieved from the medical records. After the serological diagnosis of chronic CD was confirmed using two distinct reactive serological techniques, all patients underwent an initial evaluation protocol, which included sociodemographic information (age, educational level, and race), epidemiological history of CD (transmission mode, country and region of origin, and time away from the endemic area), clinical anamnesis, and physical examination focused on chronic CD-related cardiovascular signs and symptoms, including a 12-lead electrocardiogram (ECG) and a two-dimensional Doppler echocardiogram. Based on the presence of symptoms related to the digestive form of CD, upper gastrointestinal endoscopy, esophagography, colonoscopy, and contrast barium enemas were performed. The clinical forms of chronic CD were retrospectively classified according to the 2nd Brazilian Consensus on Chagas Disease (2015), in which the cardiac form is stratified into stages A, B1, B2, C, or D, and the digestive form into megaesophagus, megacolon, or both^4^. The transmission modes of CD were categorized as follows: a) vector-borne, indicated by the history of residence in a rural area, having observed triatomines inside the home, and/or having been bitten by one; b) transfusion-related, indicated by a history of blood transfusion before 1996; c) congenital, indicated by not having a history of residence in a rural area and having a mother with CD; d) oral, indicated by not having a history of residence in a rural area or a mother with CD, along with a history of consuming açaí, sugarcane, and acerola juice in the Amazon region and/or wild game meat.

### Data analysis

Descriptive statistics are presented as means (standard deviations) for continuous variables and absolute frequencies (percentages) for categorical variables. Differences in continuous variables between transmission modes were tested using one-way ANOVA, whereas differences in categorical variables were tested using a chi-squared test. Binomial logistic regression was used to assess the association between the transmission modes and the presence of cardiac (versus non-cardiac) or digestive (versus non-digestive) forms of CD. Relative risk ratios (RRRs) from multinomial logistic regression models were estimated to evaluate associations between transmission modes and specific stages of the cardiac form (non-cardiac form vs. cardiac form without heart failure and cardiac form with heart failure) and presentations of the digestive form (non-digestive form vs. megaesophagus and megacolon). The models were fitted both unadjusted and adjusted for age, sex, educational level, and decade of admission to INI/Fiocruz (<1990, 1990-2000, 2000-2010, >2010). The significance level was set at 5%. All statistical analyses were performed using the Stata software (version 18.0; StataCorp LP., College Station, TX, USA). 

### Ethical approval

This study was approved by the INI/Fiocruz Research Ethics Committee (CAAE: 70248023.8.0000.5262) on June 30, 2023, and conducted in accordance with resolution no. 466/2012 of the Brazilian National Council of Health. The requirement for informed consent was waived due to the retrospective design of the study.

## RESULTS

**FIGURE 1:**
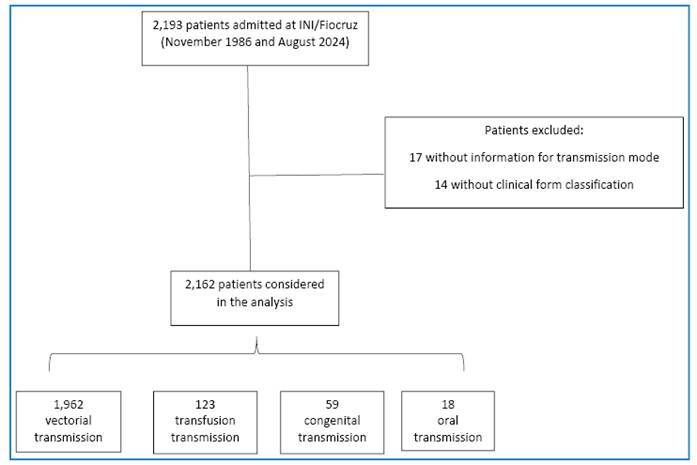
Flowchart of study participants included in the study.

Figure 1 shows the participant flowchart of this study. Out of the 2,193 individuals admitted to INI/Fiocruz between November 1986 and August 2024, 31 were excluded due to missing information on transmission mode (n=17) or clinical form of CD (n=14), resulting in an analytic sample of 2,162 patients (53.0% women; mean age 48.3 ± 12.9 years). Transmission was primarily vectorial (90.8%), followed by transfusion (5.7%), congenital (2.7%), and oral (0.8%).

The characteristics of patients stratified by transmission mode are shown in Table 1. Overall, patients with congenital or oral transmission were younger, had a lower proportion of women, self-reported Black race, and illiteracy rates, and a higher proportion were naturally from non-endemic areas. 

**TABLE 1:** Characteristics of study population stratified according to mode of CD transmission (n= 2,162).

Variables	Mode of CD transmission				p-value
	Vectorial (n=1,962)	Transfusion (n=123)	Congenital (n=59)	Oral (n=18)	
Age (years)	48.5 (12.9)	49.5 (12.0)	38.9 (10.1)	42.0 (12.4)	<0.001
Women	52.1 (1,023)	77.2 (95)	40.7 (24)	22.2 (4)	<0.001
Race					
White	48.3 (947)	58.5 (72)	44.1 (26)	55.6 (10)	0.002
Mixed	39.6 (776)	30.1 (37)	27.1 (16)	22.2 (4)	
Black	11.5 (226)	10.6 (13)	28.2 (17)	22.2 (4)	
Indigenous	0.7 (13)	0.8 (1)	0.0 (0)	0.0 (0)	
Educational level					
Illiterate	20.9 (409)	18.7 (23)	3.4 (2)	5.6 (1)	<0.001
< 9 years	60.6 (1,189)	59.4 (73)	54.2 (32)	55.6 (10)	
> 9 years	18.6 (364)	22.0 (27)	42.4 (25)	38.9 (7)	
Country of origin					
Brazil (vs other Latin American)	98.8 (1,938)	96.8 (119)	96.6 (57)	100.0 (18)	0.14
Region of origin					
Non-endemic area (vs endemic)	1.2 (23)	14.6 (18)	69.5 (41)	88.9 (16)	<0.001
Time away from endemic area					
None	3.6 (71)	2.4 (3)	1.7 (1)	5.6 (1)	<0.001
1 to 20 years	27.6 (542)	21.1 (26)	11.7 (7)	5.6 (1)	
>20 years	67.6 (1,326)	61.8 (76)	17.0 (10)	0.0 (0)	
Non endemic area	1.2 (23)	14.6 (18)	69.5 (41)	88.9 (16)	
Cardiac form					
Without cardiac form	49.8 (977)	52.0 (64)	61.0 (36)	50.0 (9)	0.80
Stage A	21.9 (428)	24.4 (30)	15.3 (9)	33.3 (6)	
Stage B1	11.9 (233)	9.8 (12)	13.6 (8)	5.6 (1)	
Stage B2	5.1 (99)	6.5 (8)	5.1 (3)	5.6 (1)	
Stage C	10.6 (207)	6.5 (8)	5.1 (3)	5.6 (1)	
Stage D	0.9 (18)	0.8 (1)	0.0 (0)	0.0 (0.0)	
Digestive form					
Without digestive form	88.4 (1,735)	88.6 (109)	93.2 (55)	88.9 (16)	0.39
Megaesophagus	6.9 (136)	8.1 (10)	1.7 (1)	11.1 (2)	
Megacolon	2.5 (48)	3.3 (4)	3.4 (2)	0.0 (0)	
Megaesophagus and megacolon	2.2 (43)	0.0 (0)	1.7 (1)	0.0 (0)	

The association between transmission modes and the presence of cardiac (versus non-cardiac) or digestive (versus non-digestive) forms of CD is presented in Table 2. No significant associations were observed between the transmission modes and the cardiac or digestive forms of CD in unadjusted and adjusted analyses. The results were similar when considering the stages of the cardiac form (non-cardiac form vs. cardiac form without heart failure and cardiac form with heart failure) and presentations of the digestive form (non-digestive form vs. megaesophagus and megacolon), in which no significant association was observed (Tables 3 and 4). 

**TABLE 2:** Odds ratio (95% CI) for the association between transmission mode and presence of cardiac and digestive clinical forms of CD.

	Cardiac Form (vs no cardiac)		Digestive Form (vs no digestive)	
	Unadjusted OR (95%CI)	Adjusted* OR (95%CI)	Unadjusted OR (95%CI)	Adjusted* OR (95%CI)
Vectorial	Ref	Ref	Ref	Ref
Transfusion	0.91 (0.63 to 1.32) p-value=0.63	0.87 (0.59 to 1.28) p-value= 0.48	0.98 (0.55 to 1.74) p-value= 0.95	1.18 (0.64 to 2.17) p-value= 0.59
Congenital	0.63 (0.37 to 1.08) p-value=0.09	1.00 (0.58 to 1.74) p-value=0.99	0.56 (0.20 to 1.55) p-value= 0.26	1.02 (0.36 to 2.93) p-value= 0.96
Oral	0.99 (0.39 to 2.51) p-value=0.99	1.40 (0.53 to 3.67) p-value = 0.50	0.96 (0.22 to 4.18) p-value=0.95	1.83 (0.40 to 8.32) p-value= 0.44

* Model adjusted for age, sex, schooling, and decade of admission at INI/Fiocruz.

**TABLE 3:** Relative risk ratio (95% CI) for the association between transmission mode and stages of cardiac form of CD.

	Stages A, B1, and B2† (cardiac without HF)		Stages C and D† (cardiac with HF)	
	Unadjusted RRR (95%CI)	Adjusted* RRR (95%CI)	Unadjusted RRR (95%CI)	Adjusted* RRR (95%CI)
Vectorial	Ref	Ref	Ref	Ref
Transfusion	1.00 (0.68 to 1.47) p-value=0.98	0.89 (0.59 to 1.32) p-value= 0.55	0.61 (0.30 to 1.25) p-value= 0.18	0.76 (0.37 to 1.60) p-value=0.48
Congenital	0.71 (0.41 to 1.24) p-value= 0.23	1.13 (0.63 to 2.01) p-value= 0.67	0.36 (0.11 to 1.19) p-value=0.09	0.57 (0.17 to 1.91) p-value 0.37
Oral	1.14 (0.44 to 2.98) p-value=0.79	1.63 (0.60 to 4.41) p-value= 0.33	0.48 (0.06 to 3.83) p-value= 0.49	0.66 (0.08 to 5.35) p-value= 0.70

† No cardiac form was used as reference. * Model adjusted for age, sex, schooling, and decade of admission at INI/Fiocruz (<1990, 1990-2000, 2000-2010, > 2010).

**TABLE 4:** Odds ratio (95% CI) for the association between transmission mode and presentation of digestive form of CD.

	Megaesophagus (n=191)^†^		Megacolon (n=98)^†^	
	Unadjusted OR (95%CI)	Adjusted* OR (95%CI)	Unadjusted OR (95%CI)	Adjusted* OR (95%CI)
Vectorial	Ref	Ref	Ref	Ref
Transfusion	0.88 (0.45 to 1.71) p-value= 0.71	1.08 (0.54 to 2.17) p-value= 0.82	0.69 (0.25 to 1.91) p-value= 0.47	0.76 (0.26 to 2.16) p-value= 0.61
Congenital	0.35 (0.08 to 1.44) p-value= 0.15	0.63 (0.15 to 2.68) p-value= 0.54	1.10 (0.34 to 3.59) p-value= 0.87	1.85 (0.55 to 6.23) p-value= 0.32
Oral	1.35 (0.28 to 5.46) p-value= 0.77	1.34 (0.51 to 10.66) p-value= 0.27	----**	----**

† No digestive form was used as reference. * Model adjusted for age, sex, schooling, and decade of admission at INI/Fiocruz (<1990, 1990-2000, 2000-2010, > 2010).

** No estimate was provided in the logistic regression model, as no patients with oral transmission presented megacolon

## DISCUSSION

Historically, vector-borne transmission of CD has been the primary route of infection. In the INI/Fiocruz cohort, the presumed classical vector-borne transmission route was predominant, with our patients (>90%) reporting an epidemiological history consistent with this transmission route. This finding aligns with those of other studies conducted in urban centers with CD patients^13^ ^,^ ^14^. Brazil halted transmission via blood products by implementing strict quality control measures in blood banks during the mid-1990s^15^. The few Brazilian studies that evaluated blood transfusion history in populations with CD identified prevalence rates between 3.3% and 7.8%, which is consistent with our findings of nearly 6%^14^ ^-^ ^16^. In Latin America, particularly in the Southern Cone region, mainly in Argentina, Bolivia, and Paraguay, congenital transmission continues to pose a significant risk for CD^7^. In Brazil, data regarding congenital transmission are limited. A systematic review covering the period from 1980 to 2013 estimated the risk of congenital transmission of *T. cruzi* to be between 0% and 5.2%, with significant geographic variability^17^. A more recent study conducted in the Federal District of Brazil estimated a transmission rate of 2.5%^18^. In our study cohort, we identified 2.7% of the patients with congenital transmission, most of whom were born in the State of Rio de Janeiro. However, the epidemiological landscape has evolved with the emergence of new modes of transmission such as oral transmission. Along with planting products, the consumption of wild game meat has also been identified as a potential source of *T. cruzi* infection. Few patients reported this transmission route (<1%), with most coming from the northern region of the State of Rio de Janeiro, a non-endemic area for CD, and associated with consuming game meat^19^. 

The diversity of transmission routes has led to different epidemiological patterns and challenges in disease control. In this setting, the relationship between specific clinical forms of CD, particularly the cardiac form, and its modes of transmission arising from a dynamic interplay between the parasite, host, and environmental factors are still poorly understood. The severity of target organ involvement in chronic CD, such as the heart, esophagus, and colon, which leads to the most severe clinical manifestations, appears to depend on multiple factors, including the host’s immune response, initial parasite load, presence of comorbidities, lifestyle habits, social determinants, access to medical services, and public policies that encourage early diagnosis and treatment as well as comprehensive care for those with the disease^20^ ^,^ ^21^. Previous studies on patients from the INI/Fiocruz cohort involving the analysis of various previously mentioned factors showed an association with a higher prevalence of the cardiac form of the disease^22^ ^,^ ^23^.

 Research on CD in urban areas indicates that the cardiac form is the most common, with a prevalence of 56%-66%. In the present study, the indeterminate form of CD was the most prevalent. The prevalence of the digestive form, whether isolated or in combination with heart disease, was consistent with the average prevalence reported in other studies. For the cardiac and cardio-digestive forms, stage A was the most common cardiac stage. Most patients in this cohort showed normal left ventricular systolic function on echocardiography, suggesting an overall favorable long-term prognosis^24^.

Very few studies have correlated transmission modes with the clinical forms of chronic CD in urban cohorts, none of which have been conducted in Brazil. A study conducted in Argentina compared the degree of cardiac involvement between two populations of patients with CD: those with permanent residence in endemic areas and those with occasional exposure to the parasite and infection through non-vectorial routes (transfusional, congenital, and others). The results showed that those with occasional exposure had a lower prevalence of the cardiac form of the disease, and when the cardiac form was present, they had a lower prevalence of dilated cardiomyopathy than those permanently residing in the endemic area. Conversely, in our study, which included a large number of patients over 40 years of age, no significant association between CD transmission modes and chronic clinical forms of CD was identified.

The retrospective cross-sectional design is an important limitation of the present study, which potentially increases the probability of measurement error and leads our results toward the null hypothesis. Moreover, the characteristics of the institution responsible for the study, as well as those of the patients who seek care there, may not necessarily reflect the clinical and epidemiological characteristics of other patients residing in its area of influence, in national endemic regions, or in other countries. Nonetheless, the large sample size of participants classified using gold-standard methods for chronic clinical forms of CD should be considered a strength of this study. Future prospective longitudinal studies are warranted to comprehensively investigate the potential association between modes of transmission and the progression of determinate clinical forms of CD.

## CONCLUSION

Vector transmission was the most common mode of transmission in patients with chronic CD, treated at an urban reference center. No significant association was found between the transmission mode and clinical forms, indicating that other mechanisms associated with the progression to chronic determinate forms should be considered.
